# Insecticide resistance intensity in *Anopheles gambiae* (*s.l*.) from five malaria epidemiological zones in Kenya

**DOI:** 10.1016/j.crpvbd.2025.100252

**Published:** 2025-03-05

**Authors:** Edith Ramaita, Silas Agumba, Joseph Mwangangi, Stanley Kitur, Lucy Wachira, Samson Otieno, David Mburu, Damaris Matoke-Muhia, Elijah Juma, Charles Mbogo, Eric Ochomo, Luna Kamau

**Affiliations:** a Ministry of Health - National Malaria Control Programme (NMCP), Nairobi, Kenya; b Centre for Global Health Research (CGHR), Kenya Medical Research Institute, Kisumu, Kenya; c Centre for Geographic Medicine Research-Coast (CGMR-C), Kenya Medical Research Institute, Mombasa, Kenya; d Centre for Biotechnology Research and Development (CBRD), Kenya Medical Research Institute, Nairobi, Kenya; e Pwani University Biosciences Research Centre (PUBReC), Mombasa, Kenya; f Pan African Mosquito Control Association (PAMCA), Nairobi, Kenya

**Keywords:** Insecticide resistance intensity, *Anopheles gambiae* (*s.l.*), Deltamethrin, Pirimiphos-methyl, Alpha-cypermethrin, Chlorfenapyr, Clothianidin

## Abstract

Insecticide-based malaria vector-control interventions have been economically important in reducing malaria prevalence. However, insecticide resistance now threatens the continued efficacy of insecticides in malaria vector control. Monitoring insecticide resistance in mosquito populations is needed to guide the implementation of effective insecticide resistance management (IRM) strategies. Thus the study assessed the levels and intensity of insecticide resistance in *Anopheles gambiae* (*sensu lato*) in five malaria epidemiological zones of Kenya, which are subjected to different vector control interventions. *Anopheles gambiae* (*s.l*.) mosquito larvae were sampled from Teso in Busia County in the lake malaria-endemic zone, Kwale in Kwale County in the coastal malaria-endemic zone, Kakuma in Turkana County, a malaria epidemic zone, Mwea in Kirinyaga County in the seasonal malaria transmission zone of Central Kenya and Thika in Kiambu County in the low-risk malaria zones. The mosquito larvae were reared into adults, and the Centre for Disease Control (CDC) bottle DD intensity of resistance assays were conducted at 2× , 5× , and 10× the discriminating doses (DD); the WHO guidelines were used to evaluate the percentage knockdown or mortality of the adult vectors. *Anopheles gambiae* (*s.l*.) mosquitoes from all the malaria epidemiological zones showed resistance to deltamethrin and pirimiphos-methyl, while mosquitoes from most of the zones were resistant to alpha-cypermethrin. However, the mosquitoes were susceptible to the other insecticides tested, i.e. chlorfenapyr and clothianidin. In cases where resistance was found, the resistance intensity ranged from low to moderate levels. *Anopheles arabiensis* was the most prevalent species in all the sites except in Busia County, where *An. gambiae* (*sensu stricto*) was the majority. The results of this study showed widespread insecticide resistance in *An. gambiae* (*s.l*.) to commonly used insecticides in different malaria epidemiological zones in Kenya. Routine surveillance of insecticide resistance through monitoring and subsequent management in the zones of occurrence is a reliable component of evidence-based policy decision-making for mitigating malaria transmission using insecticide-based vector control interventions.

## Highlights

• Insecticide resistance in *Anopheles gambiae* (*s.l.*) was studied in five malaria epidemiological zones in Kenya.
• Bioassays with deltamethrin, alpha-cypermethrin, pirimiphos-methyl, clothianidin, and chlorfenapyr were carried out.
• Results showed widespread insecticide resistance in *An. gambiae* (*s.l.*) to commonly used insecticides in the zones studied.
• Routine monitoring of resistance *via* intensity assays will help discriminate susceptible and resistant populations.
• Knowledge of the extent of resistance, vector distribution and behaviour in an area is important, as reported here.

## Graphical abstract

**Figure:**
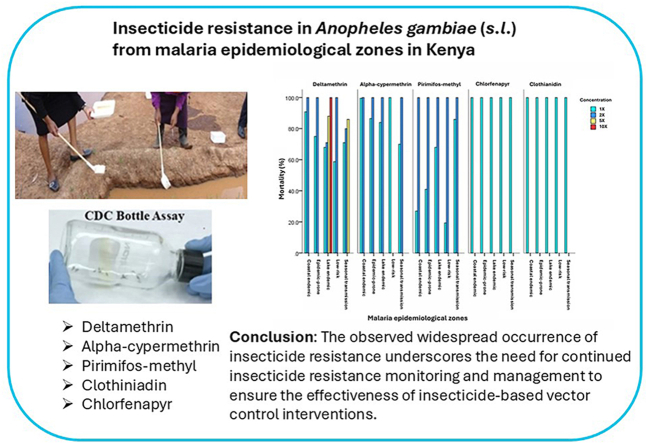

## 1 Background

Mosquito control and surveillance are crucial for malaria programmes. In sub-Saharan Africa, insecticide-treated nets (ITNs) and indoor residual spraying (IRS) with insecticides are the cornerstones of malaria vector control (Kleinschmidt et al., 2009). In Kenya, the use of ITNs and IRS, combined with case management (diagnosis and treatment of confirmed malaria cases) and intermittent preventive treatment, has significantly reduced malaria cases and fatalities (DNMP, ICF, 2021). Between 2015 and 2020, malaria prevalence decreased from 27% to 19% in the lake malaria-endemic zone, from 8% to 5% in the coastal malaria-endemic zone, and from 3% to 1% in the highland epidemic-prone zone (DNMP, ICF, 2021). Overall, the mean malaria prevalence rates in children aged six months to 14 years decreased from 8% in 2015 to 6% in 2020 (DNMP, ICF, 2021). Most of the gains in malaria control were due to the expansion of vector control interventions (WHO, 2022a). Malaria however, persists, with a disproportionate burden in sub-Saharan Africa owing to the mix of a set of efficient *Anopheles* species and *Plasmodium falciparum* (Oladipo et al., 2022) and the emergence and spread of insecticide resistance, among other factors (Bhatt et al., 2015). One significant reason for the slow progress in reducing malaria transmission is the emergence and spread of insecticide resistance in the primary mosquito vectors (Ranson, 2017). This resistance could weaken the effectiveness of chemical-based malaria control methods and threaten malaria control and elimination initiatives (WHO, 2022a)

Resistance to the four most commonly applied insecticide classes, namely organochlorines, pyrethroids, organophosphates, and carbamates, is currently on the rise across all significant vectors of malaria (https://www.irmapper.com/) (Knox et al., 2014). In Kenya, the main malaria vectors are mosquitoes of the *Anopheles gambiae* (*sensu lato*) and *Anopheles funestus* species complexes, which are also common in other parts of East Africa (Okello et al., 2006; Ogola et al., 2018; Kakilla et al., 2020). These vectors have demonstrated insecticide resistance to both the pyrethroid class which is commonly used to treat ITNs and was previously used for IRS, and the three other classes of insecticides (carbamates, organophosphates, and organochlorines) recommended for public health use (Ochomo et al., 2014; Ondeto et al., 2017; Kiuru et al., 2018).

The most comprehensive data on entomological risk come from the highly malaria-endemic lake- and coastal zones (Laurent et al., 2016; Zhong et al., 2020), and there is a paucity of insecticide resistance data across the range of malaria endemicities in Kenya. Additionally, many vector control programmes only use the diagnostic dose (1× ) of insecticides to screen for resistance, which may not reveal the extent of resistance in non-susceptible populations nor correlate with operational failure of insecticide-based vector control interventions. If, for example, two mosquito populations have a 50% mortality rate at 1× , but one achieves 100% mortality at 5× and 10× while the other remains at ∼50%, relying only on the 1× assay would mistakenly suggest their resistance is equivalent. Yet such information is important in guiding operational decisions on managing insecticide resistance (WHO, 2016). The WHO Global Vector Control Response 2017–2030 recommends that disease prevention through vector control should be informed by research and that vector surveillance and monitoring and evaluation of interventions should guide vector control (Velayudhan, 2021).

In this study, we compared the intensity of insecticide resistance to deltamethrin, pirimiphos-methyl, alpha-cypermethrin, clothianidin, and chlorfenapyr in *An. gambiae* and *An. arabiensis* at five selected sites of Kenya’s five malaria epidemiological zones, providing a representative picture of the intensity of insecticide resistance across the country. The aim was to generate data critical to guide decision-making on the rational choice of insecticide-based malaria vector control interventions that are effective and appropriate for particular settings.

## 2 Materials and methods

### 2.1 Study sites

**Fig. 1:**
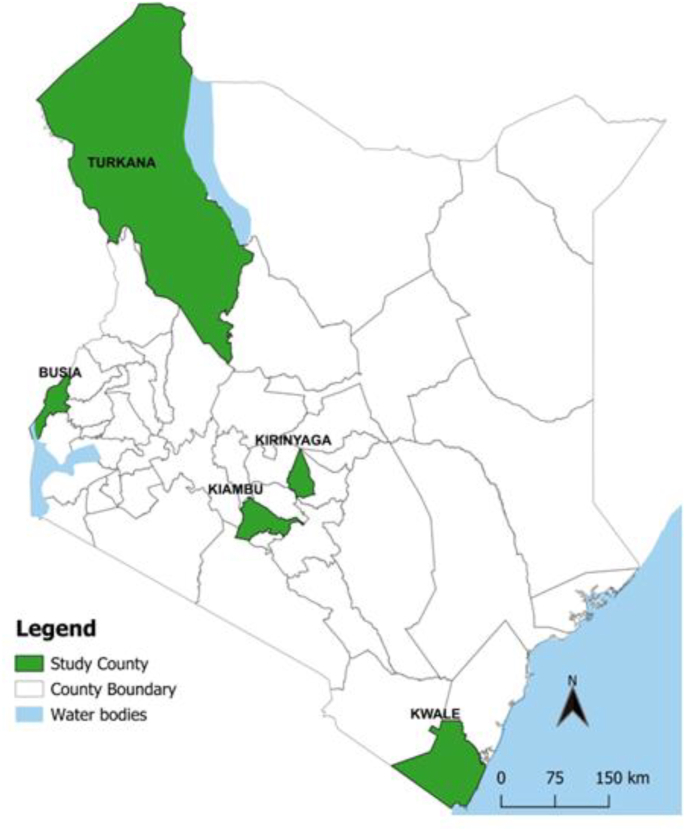
Map of Kenya showing the study areas.

Mosquitoes samples were collected from five Kenya malaria epidemiological zones (Fig. 1): Teso (0.621957, 34.346326) in Busia County in the lake malaria-endemic zone, Kwale (−4.174522, 39.452643) in Kwale County in the coastal malaria-endemic zone, Kakuma (3.746796, 34.837593) in Turkana County, a seasonal malaria-epidemic zone, Mwea (−0.382893, 37.515671) in Kirinyaga County in the seasonal malaria epidemic-prone zone and Thika (−1.248716, 36.657150) in Kiambu County in the low-risk malaria transmission zone. *Anopheles gambiae* (*s.l.*) are the main malaria vectors in these regions. These vectors occur all year round, with peak seasons during the rainy season.

### 2.2 Mosquito collection and rearing

Mosquito larvae sampling was carried out between December 2019 and February 2021. Larvae and pupae of *An. gambiae* (*s.l*.) were collected in each site from multiple breeding sites using standard dippers. Samples were pooled and transported to the KEMRI insectaries for rearing. The rearing was done at a standard temperature of 27 ± 2 °C and a relative humidity of 80 ± 10%, with larvae maintained on fine ground powder of Sera Vipan staple diet™ (Sera, Germany) fish food. Pupae were collected into cups with water and placed into 30 × 30 × 30 cm mosquito cages to maximize the collection of emerged adults. Mosquitoes were provided access to a 10% sugar solution before susceptibility testing. Adult mosquitoes were morphologically identified using identification keys for *Anopheles* spp. (Gillies and De Meillon, 1968; Coetzee, 2020).

### 2.3 Insecticide resistance intensity tests

Wheaton bottles (250 ml) were used to conduct the CDC bottle bioassays; the bottles were coated following the protocol described in CDC (2010). Mosquitoes were tested for phenotypic resistance to diagnostic doses (DD) of pyrethroids (deltamethrin 12.5 μg/ml and alpha-cypermethrin 12.5 μg/ml), organophosphate (pirimiphos-methyl 20 μg/ml), pyrrole (chlorfenapyr 100 μg/ml) and neonicotinoid (clothianidin 4 μg/ml) (WHO, 2022b). The recommended exposure time for pyrethroids and organophosphate was 30 min, with knockdown recorded at the end of the 30 min. On the other hand, the recommended exposure time for chlorfenapyr and clothianidin was 1 h, with knockdown recorded every 10 min and mortality recorded at 24 h, 48 h, and 72 h. The subset of field-collected mosquitoes from each collection site was used as a negative control and exposed to either untreated (control) papers for the WHO tube assay or acetone-treated bottle for the CDC bottle assay.

At least 100 females were used per insecticide DD, divided into at least four replicates of 20–25 females per bottle. All bottles were held vertically for the duration of the experiment. After exposure, the mosquitoes were released from the bottle into clean paper cups and provided with a 10% sugar solution soaked in cotton wool during the observation period. Confirmed resistance was defined as 90% mosquito knockdown or mortality measured at the end of exposure to the insecticide for the diagnostic period, while knockdown/mortality below 98% was indicative of possible resistance. Mosquito populations found to be resistant to the insecticides tested were exposed to higher concentrations of insecticides to provide information on the intensity of resistance. Thus, where 100% knockdown was not observed with insecticide DCs, additional testing was carried out with 2× , 5× , and 10× of the DD until 100% knockdown of mosquitoes was achieved (WHO, 2016). The modification done for the shorter exposure period for fast-acting insecticides was employed to determine the resistant population using the CDC bottle assay as it is a very sensitive technique and hence observed as the best method for insecticide resistance monitoring as highlighted in CDC (2024).

### 2.4 Species identification by PCR

A subset of mosquitoes from the insecticide resistance bioassays was randomly selected for species identification using the polymerase chain reaction (PCR) as highlighted in Scott et al. (1994) to distinguish between members of the *An. gambiae* species complex. DNA was extracted from mosquito wings and legs following the alcohol precipitation technique, as described in Scott et al. (1993). The amplicons were electrophoresed in a 3% agarose gel stained with ethidium bromide. The sibling species were visualized and scored against size markers under ultraviolet illumination.

### 2.5 Data analysis

The efficacy of the insecticides against wild *Anopheles* spp. was calculated as percentage knockdown (KD) or mortality, following the World Health Organization guidelines (WHO, 2016) on insecticide susceptibility: KD/mortality ≥ 98% indicates susceptibility, KD/mortality < 90% indicates the existence of resistance, and KD/mortality between 90% and 97% represents suspected resistance that needs confirmation. For higher concentrations of insecticides, exposure at the 2× the DD is recommended in the series of concentrations for the intensity of resistance with the bottle bioassay to indicate whether screening with the 5× the DD is necessary and would be indicative of low intensity resistance for mortality ≥ 98% to be confirmed by results of exposure at the 5× DD (CDC, 2024). Knockdown ≥ 98% at the 5× DD indicates a low resistance intensity; hence, testing with a higher concentration (10× the DD) is recommended. Knockdown ≥ 98% at the 10× DD confirms moderate resistance intensity, while mortality < 98% at the 10× DD indicates high resistance intensity.

## 3 Results

### 3.1 Sample size and species composition

A total of 6102 adult female *An. gambiae* (*s.l*.) F_0_ were used for the susceptibility bioassay from the five Kenyan malaria epidemiological zones. These included the lake malaria-endemic zone (*n* = 1457), the coastal malaria-endemic zone (*n* = 1950), the malaria epidemic-prone zone (*n* = 1022), the malaria low-risk zone (*n* = 788), and the seasonal malaria transmission zone (*n* = 885). The PCR analysis revealed that in the lake malaria-endemic zone, the dominant *Anopheles* species were *An. gambiae* (*s.s.*) at 53% and *An. arabiensis* at 47%. In the malaria low-risk zone, 96% of *An. arabiensis* and 4% of *An. gambiae* (*s.s.*) were identified. Mosquitoes from the coastal endemic, seasonal transmission and epidemic-prone zones were all *An. arabiensis*.

### 3.2 Insecticide susceptibility bioassays

Resistance to deltamethrin was observed in all epidemiological zones, with the highest level being observed in Busia, the lake malaria-endemic zone, where the samples assayed were only susceptible at 10× the DD (Table 1). The knockdown rate for deltamethrin in the seasonal transmission zone was 86% when exposed to 5× the DD, indicating a high resistance intensity. However, full susceptibility to deltamethrin at 2× the DD was observed for *An. gambiae* (*s.l*.) from Turkana, Kiambu and Kwale indicating low resistance intensity. For alpha-cypermethrin, only *An. gambiae* (*s.l*.) from the Kiambu was susceptible to the DD, while there was full susceptibility at 2× DD in all the other sites. *Anopheles gambiae* (*s.l.*) populations from all the malaria epidemiological zones were susceptible to pirimiphos-methyl (2× DD), chlorfenapyr (1× DD), and clothianidin (1× DD) (Table 1, Supplementary file S1).

**Table 1:** Susceptibility of *Anopheles gambiae* (*s.l*.) from different malaria epidemiological zones in Kenya to commonly used public health insecticides.

Malaria epidemiological zone (County)	Insecticide	Dose	%KD/mortality (*n*)	95% CI
Lake malaria-endemic zone (Busia)	Alpha-cypermethrin	1×	84 (387)	81.4–92.1
		2×	100 (89)	100–100
	Deltamethrin	1×	68 (100)	61.0–75.1
		2×	71 (100)	65.6–77.5
		5×	88 (100)	85.0–91.2
		10×	100 (48)	100–100
	Pirimiphos-methyl	1×	68 (272)	60.6–79.7
		2×	100 (72)	100–100
	Chlorfenapyr	1×	100 (94)	100–100
	Clothianidin	1×	100 (106)	100–100
Low-risk zone (Kiambu)	Alpha-cypermethrin	1×	100 (67)	100–100
	Deltamethrin	1×	58.8 (170)	55.2–92.1
		2×	100 (100)	100–100
	Pirimiphos-methyl	1×	19.4 (124)	0–45.8
	Chlorfenapyr	1×	100 (127)	100–100
	Clothianidin	1×	100 (100)	100–100
Seasonal transmission zone (Kirinyaga)	Alpha-cypermethrin	1×	70 (82)	60.2–78.8
		2×	100 (91)	100–100
	Deltamethrin	1×	71 (86)	60.6–82.2
		2×	80 (103)	74.6–84.5
		5×	86.0 (125)	97.0–101.4
	Pirimiphos-methyl	1×	86 (94)	83.6–88.8
		2×	100 (100)	62.3–89.7
	Chlorfenapyr	1×	100 (71)	100–100
	Clothianidin	1×	100 (104)	100–100
Coastal endemic zone (Kwale)	Alpha-cypermethrin	1×	99.5 (200)	95.8–102.2
	Deltamethrin	1×	90.8 (500)	80.7–95.3
		2×	100 (100)	100–100
	Pirimiphos-methyl	1×	27.0 (200)	22.8–33.2
	Chlorfenapyr	1×	100 (375)	100–100
	Clothianidin	1×	100 (375)	100–100
Epidemic-prone zone (Turkana)	Alpha-cypermethrin	1×	86.5 (260)	86.4–97.6
		2×	100 (88)	100–100
	Deltamethrin	1×	75.0 (273)	71.7–88.6
		2×	100 (51)	20.5–149.5
	Pirimiphos-methyl	1×	41.0 (105)	9.8–66.0
		2×	100 (56)	100–100
	Chlorfenapyr	1×	100 (100)	100–100
	Clothianidin	1×	100 (89)	100–100

*Abbreviations*: CI, confidence interval; KD, the percent of *An. gambiae* (*s.l.*) knockdown during the exposure period (alpha-cypermethrin, deltamethrin and pirimiphos-methyl); *n*, the number of *An. gambiae* (*s.l.*) exposed during the bioassay.

For the lake endemic and the low-risk malaria epidemiological zones where both species were found, *An. gambiae* (*s.s*.) and *An. arabiensis* were further stratified by resistance status to allow a comparison of resistance levels between the species deltamethrin at the DD (1× ). In the lake malaria-endemic zone, where both species were present in comparable proportions, a significantly higher proportion of *An. gambiae* (*s.s.*) survived insecticide exposure relative to *An. arabiensis* for deltamethrin (*z* = 4.4, *P* < 0.0001), alpha-cypermethrin (*z* = 2.6, *P* < 0.0091) and pirimiphos-methyl (*z* = 4.2, *P* < 0.0001), respectively (Table 2). None of the three *An. gambiae* (*s.s*.) mosquitoes identified from the low-risk zone were knocked down at the DD (1× ) for deltamethrin.

**Table 2:** Insecticide resistance levels between sibling species in the lake malaria-endemic zone (Busia County).

Insecticide/Species	*An. gambiae* (*s.s*.)	*An. arabiensis*
	% alive	% alive
Deltamethrin (1× )	38.2 (55)	2.2 (46)
Pirimiphos -methyl (1× )	45.9 (37)	4.9 (41)
Alpha-cypermethrin (1× )	60.0 (35)	34.4 (90)

## 4 Discussion

Insecticide resistance surveillance is a key pillar of the WHO Global Vector Control Response (GVCR) plan (WHO and UNICEF, 2017). Such surveillance is a basic requirement for developing a rational and subsequent use of insecticide resistance management plan (IRM) that guides the selection of insecticides for vector control. The results of the present study have shown that *An. arabiensis* is the most dominant species in sampled areas in all the epidemiological zones of Kenya except in the lake malaria-endemic region of Busia where *An. gambiae* (*s.s.*) predominated. Previous studies reported that *An. gambiae* (*s.s.*) was the dominant subspecies in many regions of the country. However, a shift in the composition of malaria vectors has been documented, coinciding with the widespread use of ITNs in many regions of the country (Bayoh et al., 2010; Hemingway, 2014; McCann et al., 2014). Routine surveillance for insecticide resistance is crucial for managing resistance levels and preventing operational failure.

Resistance to deltamethrin, the commonly used pyrethroid insecticide, was observed in *An. gambiae* (*s.l*.) from across the four epidemiological zones: Busia County in the lake malaria-endemic zone; Kirinyaga County within the seasonal malaria transmission zone; Turkana County in the malaria epidemic-prone zone; and Kiambu County within the low-risk zone. Additionally, a high possibility of resistance in the coastal endemic zone (Kwale) required confirmation, having shown a mortality of 90.8%. Further, insecticide resistance to the pyrethroid alpha-cypermethrin was also observed in *An. gambiae* (*s.l*.) from Busia, Kirinyaga and Turkana, while mosquitoes from Kwale and Kiambu were susceptible. The observed pyrethroid resistance corroborates the previously reported findings of widespread insecticide resistance in the country and sub-Saharan Africa in general (http://www.irmapper.com) (Knox et al., 2014). In western Kenya, the lake malaria-endemic zone, where Busia is located, showed a reduced knockdown to pyrethroids in malaria vectors following the distribution of ITNs in 2010 as reported in previous studies (Ochomo et al., 2014; Ondeto et al., 2017). The wide-scale implementation of the pyrethroid-based ITNs and IRS vector control tools in malaria-endemic western and coastal regions is likely the major contributor underlying the selection pressure for the vectors to select for resistance (Ondeto et al., 2017). The selection pressure to pyrethroid resistance could be further attributed to the exposure of mosquitoes to agrochemicals used for crop protection (Diabate et al., 2002; Mueller et al., 2008; Nkya et al., 2014).

The higher resistance in *An. gambiae* (*s.s.*) from Busia in the lake malaria-endemic region is likely due to its high propensity for feeding on humans and resting inside human dwellings after blood-feeding, compared to *An. arabiensis*, a species with a more varied feeding and resting behaviour (Kitau et al., 2012; Munywoki et al., 2021). The adaptability of *An. arabiensis*, in terms of blood-feeding behaviour, reduces the possibility of contact with insecticide-treated surfaces, lessening the likelihood of resistance. This adaptability also affects the abundance of *An. gambiae* (*s.s.*) relative to *An. arabiensis* in areas using ITNs, as seen in western Kenya (Bayoh et al., 2010). The findings of higher levels of resistance in *An. gambiae* (*s.s*.) compared to *An. arabiensis* in the present study is in concordance with observations of higher frequencies of the knockdown (kdr) mutation in populations of *An. gambiae* (*s.s.*) within the lake malaria-endemic region (Ochomo et al., 2015; Wanjala and Kweka, 2018). These findings suggest that the efficacy of the insecticides deployed in the insecticide-based vector control tools, such as ITNs and IRS targeting indoor spaces for which resistance was observed, may have been compromised.

With pyrethroid resistance in malaria vectors threatening the efficacy of the standard ITNs, piperonyl-butoxide (PBO) synergist nets combined with pyrethroid have shown enhanced potency against resistance mediated by mono-oxygenase mechanisms and is a strategy for combating resistance (Martin et al., 2021). Another approach to the rational management of insecticide resistance is to rotate insecticide classes previously used with one having a different mode of action. In the present study, *An. gambiae* (*s.l*.) mosquitoes from all the sites sampled were susceptible to clothianidin and chlorfenapyr, and these new insecticide classes may provide the solution to the management of resistance through the switching approach. Clothianidin and chlorfenapyr have now been recommended for use by WHO in areas with confirmed pyrethroid resistance and may be deployed singly or in combinations with other insecticides. Clothianidin, either as a sole active ingredient or in combination with deltamethrin, has been prequalified for use in vector control in IRS in Kenya (IRS implementation strategy 2020–2024). The Kenya IRM plan focuses on the three vector control strategies, i.e. ITNs, IRS, and larval source management (LSM), deployed according to malaria risk stratification. Innovations, especially those that address the emerging threat of insecticide resistance and modern, effective malaria vector control methods, are considered as they become available. All vector control interventions can be deployed in the context of integrated vector management (IVM) (DNMP, ICF, 2021).

The WHO also recommends the use of pyrethroid-chlorfenapyr ITNs in areas with pyrethroid resistance. An example is the new Interceptor® G2 (IG2), which combines chlorfenapyr with the pyrethroid alpha-cypermethrin, which has been demonstrated to provide residual control of pyrethroid-resistant malaria vectors in IRS (Ngufor et al., 2020). Thus, chlorfenapyr offers a promising future for both IRS and insecticide-treated net-based vector control interventions. The susceptibility of malaria vectors to the organophosphate insecticide pirimiphos-methyl across all the selected sites in the epidemiological zones suggests that it can be used as an alternative in managing pyrethroid resistance in the study areas. However, the resistance in malaria vectors to organophosphates has been attributed to the overexpression of esterase enzymes not linked to any specific site of action or alteration of acetylcholinesterase (AChE) due to a single glycine to serine amino-acid substitution at position 119 of the AChE gene in another site (Weill et al., 2004). Except for the western region, where pirimiphos-methyl has been used for IRS to supplement the pyrethroid-based ITNs (Abong’o et al., 2020), this insecticide has not been used in any of the other study regions.

The proportions of *An. gambiae* (*s.s.*) and *An. arabiensis* in the samples from Busia that were analyzed were not significantly different. This is unlike the studies conducted between 2012 and 2014, in which an overall higher proportion (about 77.2% in larval collections) of *An. gambiae* (*s.s.*) was found (Githinji, 2020). Shifts in relative proportions of different vector species over time have been reported; in western Kenya, for example, there has been a steady decline in the proportions of the more endophilic and endophagic *An. gambiae* (*s.s.*) relative to *An. arabiensis* in an area of high coverage of ITNs was reported (Bayoh et al., 2010). The ownership and use of ITNs in Busia has recently been estimated to be over 90% (Ng’ang’a et al., 2021). This high coverage may partially explain the observed changes in mosquito population structure. For Thika in Kiambu, the present study reported *An. arabiensis* as the dominant species of the *An. gambiae* (*s.l*.) species complex, accounting for 96.2% of the samples analyzed. The predominance of *An. arabiensis* in this urban setting may be attributable to the ability of the species to adapt to polluted environments as previously reported by Jones et al. (2012) and Azrag and Mohammed (2018), factors that may also account for its predominance in the Turkana study site.

## 5 Conclusions

This study reported phenotypic resistance to commonly used public health pyrethroids (deltamethrin and alpha-cypermethrin), pirimiphos-methyl, clothianidin and chlorfenapyr insecticides in malaria vectors from selected sites across Kenyan epidemiological zones. The high insecticide resistance in the lake malaria-endemic region underscores the need for constant surveillance using available monitoring tools and deploying rotation of vector control tools. These findings highlight the need for regular entomological surveillance and monitoring of insecticide resistance and for investment in new vector control strategies that can supplement or even replace the use of synthetic insecticides. An understanding of insecticide resistance and vector bionomics is thus key in guiding the deployment of vector control interventions for maximum impact. In addition to managing insecticide resistance, it is also critically important to deploy Integrated Vector Management (IVM) strategies to ensure that vector species such as *An. arabiensis*, which has outdoor resting and feeding tendencies, has been reached to prevent the possibility of residual malaria transmission by such species in areas with ITNs scale-up.

## CRediT authorship contribution statement

**Edith Ramaita:** Methodology, Investigation, Data curation, Writing – original draft, Writing – review & editing. **Silas Agumba:** Methodology, Investigation, Data curation, Writing – original draft, Writing – review & editing. **Joseph Mwangangi:** Conceptualization, Methodology, Investigation, Writing – review & editing. **Stanley Kitur:** Methodology, Investigation, Writing – review & editing. **Lucy Wachira:** Methodology, Investigation, Writing – review & editing. **Samson Otieno:** Methodology, Writing – review & editing. **David Mburu:** Conceptualization, Writing – review & editing. **Damaris Matoke-Muhia:** Conceptualization, Methodology, Data curation, Writing – review & editing. **Elijah Juma:** Conceptualization, Investigation, Writing – review & editing. **Charles Mbogo:** Conceptualization, Writing – review & editing. **Eric Ochomo:** Conceptualization, Methodology, Investigation, Data curation, Writing – review & editing. **Luna Kamau:** Conceptualization, Methodology, Investigation, Data curation, Writing – original draft, Writing – review & editing.

## Ethical approval

This study was approved by the KEMRI Scientific and Ethics Review Unit (SERU), (protocol approval no. KEMRI/SERU 3854), and the KNH-UoN Ethics and Research Committee (protocol approval no. P851/11/2022). This research was licensed by the National Commission for Science, Technology, and Innovations (NACOSTI) (license no. NACOSTI/P/23/24392).

## Funding

The study was supported by the 10.13039/100000865Bill and Melinda Gates Foundation Award ID OPP1210319 to Luna Kamau.

## Declaration of competing interests

The authors declare that they have no known competing financial interests or personal relationships that could have appeared to influence the work reported in this paper.

## Data Availability

All data generated during the study and supporting the conclusions of this article are included within the article and its supplementary file.

## Appendix A Supplementary data

Supplementary file S1. Row data.
